# A Comparison of the Shear Bond Strength of Orthodontic Brackets Bonded With Different Orthodontic Adhesives

**DOI:** 10.7759/cureus.39115

**Published:** 2023-05-16

**Authors:** Shikha Shalini, Anupriya Jha, Panchali Kashyap, Priyanka Gupta, Shubhangi Rajbhoj, Sayali Bhandari

**Affiliations:** 1 Department of Orthodontics and Dentofacial Orthopaedics, Smitam Arogyam Dental Centre, Patna, IND; 2 Department of Pedodontics and Preventive Dentistry, Shyamal Hospital, Patna, IND; 3 Department of Public Health Dentistry, Lakhimpur Medical College and Hospital, Assam, IND; 4 Department of Pediatrics and Preventive Dentistry, Index Institute of Dental Sciences, Indore, IND; 5 Department of Periodontology, Sinhgad Dental College and Hospital, Pune, IND; 6 Department of Pedodontics and Preventive Dentistry, ACPM Dental College, Dhule, IND

**Keywords:** self etch, enamel bonding, adhesive remnant index, shear bond strength, orthodontic brackets

## Abstract

Aim: The purpose of this study was to examine the strength of the bonding between orthodontic brackets and different orthodontic adhesives.

Materials and methods: To achieve this, the researchers selected 120 extracted premolars and divided them into four groups randomly. Then, one of the three adhesives, Transbond XT, Bracepaste, or Heliosit, was used to join the brackets together. After bonding, the force needed to remove the brackets was tested, and the amount of adhesive that remained on the tooth surface was also noted (referred to as the adhesive remnant index or ARI).

Results: The results showed that Transbond XT had an average bond strength of 18.05 ± 5.6 MPa, Bracepaste had an average bond strength of 16.6 ± 5.1 MPa, and Heliosit had an average bond strength of 16.2 ± 4 MPa. The average bond strength and ARI scores for Transbond XT and Bracepaste were similar at 11.10 MPa. The study found that the light-cured composite adhesives provided the strongest bond and left the tooth surface smoother and cleaner.

Conclusion: In conclusion, the study presented significant information about the impact on the enamel surface as well as the strength of the bond between orthodontic brackets and different adhesives.

## Introduction

The adhesive system's capacity to withstand stresses placed on the junction of the bracket, adhesive, and enamel is crucial to the success of fixed orthodontic appliance therapy. When assessing the bonding material's strength, shear bond strength (SBS) is a critical component to take into account. Since Buonocore first introduced the acid-etch bonding process in 1955, the bonding of orthodontic attachments using light-cured adhesives has represented a significant advancement in the field of orthodontics [1]. It is acknowledged that Newman's invention of enamel bonding in 1979 represented a significant advancement in the field of orthodontic care [2]. Tavas and Watts were the first to discuss the use of light-cured materials for orthodontic bonding in 1979 [3]. About 5.9 MPa to 7.8 MPa of SBS are needed to withstand masticatory forces [4].

This study compared the SBS of various orthodontic adhesives that were applied to bond orthodontic brackets.

## Materials and methods

The study was conducted at the Department of Orthodontics and Dentofacial Orthopaedics, Buddha Institute of Dental Sciences and Hospital, Patna. Ethical clearance was obtained from the Buddha Institute of Dental Sciences and Hospital ethical committee with institutional reference number 650/BIDSH, and consent was taken from the participants before sample collection. SBS was assessed at the National Institute of Engineering and Technology in Patna.

One hundred and twenty human maxillary first premolars, which were extracted for orthodontic treatment, were taken for the study. Inclusion criteria include teeth that must be vital at the time of extraction and teeth that must be fully erupted in the oral cavity with a sufficient clinical crown. Exclusion criteria include teeth that must not have any developmental defects, there should be no cracks or fractures present on the tooth during extraction, teeth that must not be carious or restored at the time of extraction, and teeth surfaces that must not have been previously treated with a chemical agent.

To stop dehydration and bacterial growth, the collected teeth were rinsed and put in a 0.1% thymol solution until required, a period not exceeding four weeks. One hundred and twenty extracted upper premolars were randomly divided into four groups of 30 each into four different groups of bonding materials: Group I, Group II, Group III, and Group IV, as described in Table 1.

**Table 1:** Four different groups of bonding materials

Group-I	N-30	Conventional Etch +Light Cure (Transbond XT) Adhesive
Group-II	N-30	Conventional Etch + Light Cure (Bracepaste) Adhesive
Group-III	N-30	Self Etch Primer (TransbondPlus) + Light Cure (Transbond XT) Adhesive
Group-IV	N-30	Conventional Etch + Single Component Light Cure Adhesive

Bracket description: The brackets used in this study were stainless steel (Whiteroot O2 bracket system, India) upper first and second premolar brackets with a slot dimension of 0.22" x 0.28." They were of MBT prescription and had a torque of -7 degrees and an angulation of 0 degrees. The bracket base surface area was 10.73mm², and the mesh size was 80 gauge.

Etching agent: Total etch agent (Ivoclar Vivadent, India), which contains 37% phosphoric acid, was used for Group I, Group II, and Group IV.

Primer: Transbond XT primer from 3M Unitek was used for Group I and Group II, while Transbond Plus self-etch primer from the same manufacturer was used for Group III.

Bonding adhesives: In Group I, Transbond XT light cure adhesive from 3M Unitek was used to bond the brackets. In Group II, Bracepaste light cure adhesive from American Orthodontic was used. In Group III, Transbond Plus self-etching primer and Transbond XT light cure adhesive from 3M Unitek were used as adhesives to bond the brackets. Heliosit single-component light cure adhesive from IvoclareVivadent was used to bond the brackets in Group IV.

Methods of application

Sample preparation: Each tooth was embedded in a block of cold-cure acrylic material, which was then stored in water. The facial surface of the teeth was cleaned and polished with pumice and rubber prophylactic cups.

Bonding Procedures: All brackets were bonded to the teeth using the standard bonding techniques, where curing was done between 5 to 30 seconds. Materials were handled in accordance with the manufacturer's instructions throughout the entire bonding process. Group I, Group II, and Group IV samples were treated with 37% phosphoric acid for 15 seconds, washed with distilled water, and dried. Transbond XT primer was applied to the etched enamel tooth surface of Group I and Group II samples. Transbond XT light cure adhesive and Bracepaste light cure adhesive were used for Group I and Group II, respectively. In Group III, a thin coat of Transbond Plus self-etch primer was applied and light-cured for 10 seconds. Transbond XT light cure adhesive resin was applied in the same manner as Group I and Group II. Heliosit single-component light cure adhesive was then applied to the bracket base, and the adhesive was cured for 20 seconds each on the cervical and incisal surfaces of the bracket using a BluephaseN light cure unit from Ivoclare Vivadent.

Methods of evaluation

Evaluation of SBS: The samples after bonding were stored in deionized distilled water at 37°C for 24 hrs. After 24 hrs, samples were taken for evaluation of SBS. The samples were secured in a clamp device. The samples were then stressed in a gingivo-incisal direction for a shear bond test on the Instron universal testing machine (Zwick Z250), according to a similar procedure described in the literature by Rajagopal et al. [5].

During each test, the cross-head speed of the Instron Machine was moved at a constant speed of 1 mm per minute [6]. The maximum stress necessary to debond or initiate bracket fracture was recorded in Newtons and then calculated by dividing the debonding force by the bracket base surface area yielding megapascals (MPa) as a unit.

All data was collected and sent for statistical analysis. The mean SBS of the four groups was compared by one-way analysis of variance (ANOVA).

## Results

The mean SBS (MPa) of Group 1 is 18.05 (range 7.9-38.1), the mean value of Group II is 16.6 (range 8.3-24.1), the similar mean value of Group III and IV is 16.2 and 7.3, respectively, with a range of 8.1-29.9 and 3.2-15.7. One-way ANOVA showed a statistically significant difference in SBS among various groups (P<0.001) as shown in Table 2.

**Table 2:** Mean SBS value (Mpa) among different groups of study SBS: shear bond strength, SD: standard deviation

Group	SBS (MPa) Mean	SD	Range	p-value
Group I	18.05	±4.2	7.9-38.1	0.001
Group II	16.6	±4.9	8.3-24.1	
Group III	16.2	±4	8.1-29.9	
Group IV	7.3	±3.2	3.2-15.7	

## Discussion

The aim of this study was to ensure a minimal deviation from mean values by selecting 30 teeth per group. In 1994, Fox et al. suggested that in vitro bond strength test results can be considered accurate for samples containing 20-30 specimens [7]. The selection criteria for teeth in this study were similar to those used by Bishara et al. [8] and Scougall Vilchis et al. [9]. Saline was used to gently remove soft tissue attachments, blood, and saliva from extracted premolars and then stored in 0.1% (wt/vol) thymol to inhibit bacterial growth. Thymol at a concentration of 0.1% (wt/vol) has also been used by other researchers such as Bishara et al. [10,11] and Naidu et al. [12] for teeth storage.

In Group I, the traditional etch method outlined by Bishara et al. was combined with the Transbond XT adhesive. Group II followed the same steps but applied Bracepaste [10]. In Group III, a two-step bonding process similar to that used in earlier studies by Bishara et al. [10], Sirirungrojying et al. [13], and Vicente et al. [14] was used with the Transbond Plus self-etch primer. Similar to earlier studies by Tecco et al. [15] and D'Attilio et al. [16], Group IV was sealed with a low-viscosity adhesive called Heliosit right away following etching with 37% phosphoric acid.

While some researchers have used a tensile mode, the bond strength assessment was carried out in a shear mode. Bond strength was tested using tension, torque, and shear loads in a preliminary study by Mascia and Chen in 1982, and they discovered that only the shearing mode produced reliable results [17]. The shear mode better simulates the clinical situation because the forces applied to brackets in vivo are not likely to be solely tensile. In order to apply consistent shear forces across all samples for this study, teeth were clamped in an Instron Universal testing machine (Zwick Z250) with a chisel placed on the incisal side of the bracket and clamped to a self-centering device. Using a shear bond test on the Instron machine, the specimens were stressed in an incisal-gingiva direction until failure occurred, following a similar process described by Owens and Miller [18].

The SBS of four different adhesives, including Transbond XT, BracePaste, Transbond Plus with Transbond XT, and Heliosit, were evaluated and analyzed statistically. The results showed that the mean SBS of Group I was 18.05 ± 5.6 Mpa, followed by Group II with 16.6 ± 5.1 Mpa, Group III with 16.2 ± 4 Mpa, and Group IV with 7.3 ± 3.2 Mpa. Group I had the highest mean SBS, followed by Group II, Group III, and Group IV. The mean SBS of Group I was found to be the highest among all groups with a value of 18.05 Mpa.

In a study by Scougall Vilchis et al. [9], the SBS of various adhesives, including Transbond XT and Transbond Plus, was compared. Transbond XT had the highest SBS of 19.0 MPa, followed by Transbond Plus and three other self-etching adhesives, according to the results. These results are in line with our investigation. However, the Transbond XT SBS reported in Owens and Miller's study [18] was only 7.9 MPa, which is significantly less than in our study. The methodological differences used by Owens and Miller to etch the teeth for 30 seconds, store the samples in 10% formalin, and light-cure at 450 nm [18] which has an added advantage.

Rajgopal et al. reported a higher SBS of Transbond Plus (11.104 Mpa) than Transbond XT (9.54 Mpa) [5]. However, our research findings were not consistent with Rajgopal's study [5], which may be attributed to differences in the load of the UTM machine used to test SBS. Several other studies have compared the SBS of Transbond XT with flowable composite, including Uysal et al. [19] and D'Attilio et al. [16], who reported SBS values of 25.5 Mpa, 23.47 Mpa, and 23.23 Mpa, respectively, which are higher than the values obtained in our research. These differences may be due to variations in storage time and debonding procedures employed. In our study, we also compared the SBS of Bracepaste (16.6 Mpa), cured using the conventional technique, with that of Transbond XT (16.6 Mpa), and found no significant difference in the values obtained. Notably, Bracepaste's SBS was higher than the minimum values recommended by Reynolds [4] for clinically effective bonding, and it was the second highest in our study. The FDA in the USA compared the SBS of Bracepaste (29.66 Mpa) with Transbond XT (30.62 Mpa) in 2016 and found that BracePaste Adhesive performs as well as Transbond XT, and any slight differences do not affect the original function or intended purpose of the adhesive. Finally, when comparing the SBS of Bracepaste (16.6 Mpa) or conventional etch technique (17.5 Mpa) with Heliosit (7.3 Mpa), a significant difference in the values was observed.

Our study's findings show that Group III (Transbond Plus self-etch primer with Transbond XT adhesive) had a 16.2 Mpa SBS. Similar to this, Transbond Plus with Transbond XT had a 16.6 Mpa SBS, according to Scougall Vilchis et al. [9]. The self-etch primer had a clinically acceptable bond strength of 11.10 Mpa, according to Rajagopal, who conducted a comparison study between conventional, self-etch, and moisture-insensitive primers [5]. The different curing times might have played a role in this result. The SBS of two self-etch primers (Transbond Plus and First Step) was assessed and contrasted with the control (Transbond XT) in a study conducted by Trites et al. in 2004 [20]. Transbond XT, the control, had an SBS of 12.71 Mpa, followed by Transbond Plus (10.96 Mpa) and First Step (5.30 Mpa), according to the researchers. The significant difference in SBS may have been caused by the crosshead speed (2 mm per minute) of the jig attached to the UTM machine.

Heliosit, which was used in this study, had the lowest SBS, measuring 7.3 Mpa. In comparison to Bracepaste, TransbondXT, and TransbondXT with Transbond Plus, Heliosit had a significantly lower bond strength. The SBS of Heliosit was within the range Reynolds [4] had suggested as ideal in a prior investigation. Additionally, Pickett et al. reported that the bond strength of in vivo tests was significantly lower than that of in vitro tests [21]. The results of the current study for the bond strength of Heliosit were comparable to those obtained by Bradburn and Pender [22] but significantly inferior to those obtained by Schmidlin et al. [23]. The mean SBS of Heliosit adhesive varied significantly between studies, suggesting that inconsistent study designs may make it difficult for researchers to compare results between studies.

Even when the same material is used in different studies, variations in operator techniques and methodologies, such as storage time before debonding, thermocycling, debonding device, bonding area, and variations in bracket mesh, can cause differences in bond strengths. As a result, it is not always possible to compare bond strength values numerically, and studies on bond strength are primarily significant for their relative values. The disparity in mean SBS among the cited studies suggests that each study needs its own control. In addition to testing the SBS, a study was done to determine how adhesives affected the enamel surface after debonding. The goal of this study was to find an adhesive that had the best possible SBS while also being gentle on the tooth's enamel surface after debonding.

Assessment of site of fracture

Researchers have used the adhesive remnant index (ARI), which was created by Artun and Berglund, to standardize bond failure analysis [24]. The ARI allows for statistical analysis and cross-study comparisons, despite the fact that it may oversimplify the complicated bond failure analysis-related issues. The SBS and ARI values can be used to correlate the results from scanning electron microscopy (SEM). When the conditioner affected the enamel surface more severely, stronger bonds and more adhesive remnants were seen. On the other hand, softer etch patterns had lower mean SBS and ARI scores. Although the Heliosit-treated enamel surface was harmed, no adhesive remnants were visible. After bracket debonding, the enamel surfaces of Group I, Group II, and Group IV surfaces underwent acid etching, and under SEM, these surfaces appeared porous. As seen in Group III, self-etching primer-treated enamel had smoother and cleaner surfaces. Bond strength is impacted by stresses that are challenging to measure and quantify precisely, such as torsion, tensile, shear, or a combination of these forces. Although defining a threshold for SBS in vivo would be advantageous, a number of barriers might stand in the way of this. Therefore, based on their own clinical judgment and the available research, clinicians must choose which kind of adhesive to use. Although the results of this study show that self-etching primers (Transbond Plus with Transbond XT) and light-cure composite adhesives (Transbond XT, Bracepaste) are stronger, Transbond Plus with Transbond XT had the best SBS and also provided a smoother and cleaner enamel surface after debonding.

The study has limitations, including the need for more research in this field given the conflicting results reported in the literature and the difficulty of standardizing in vitro testing for biomaterials. Additionally, in vivo studies are required to verify the findings of the current study. Additional research is needed to determine the SBS of these self-etching primers under in vivo settings, such as SEM analysis of a few surfaces from each group, in order to further validate the findings, which could replace the conventional acid etching primer adhesive technique.

## Conclusions

The results of the current study indicate that Group I, which was bonded with Transbond XT, had the highest SBS, followed by Group II, which was bonded with Bracepaste, Group III, which was bonded with Transbond Plus with Transbond XT, and Group IV, which was bonded with Heliosit had the lowest SBS.

Furthermore, while the majority of the adhesive in Groups I and II remained on the tooth, indicating failure at the bracket-adhesive interface, the majority of the adhesive in Groups III and IV remained on the bracket, indicating failure at the enamel-adhesive interface. In Group I, Group II, and Group III, a significantly significant association was discovered between SBS and ARI score.
